# Tumeur à cellules géantes des os du carpe, localisation rare

**DOI:** 10.11604/pamj.2015.21.135.6892

**Published:** 2015-06-17

**Authors:** Ismail Hmouri, Ahmed Elbardouni

**Affiliations:** 1 Clinique de Chirurgie Orthopédique, CHU avicenne, Rabat, Maroc

**Keywords:** Tumeur, carpe, benigne, Tumor, carpal bones, benign

## Image en medicine

Il s'agit d'une jeune fille de 19 ans, sans antécédents pathologiques notables, qui consulte pour des douleurs du poignet gauche d'allure inflammatoire, avec perte de la force de préhension, sans déficit vasculo-nerveux, évoluant pendant 2 ans. Le bilan radiologique (A) a montré des images lacunaire intéressant le scaphoïde carpien, le trapèze, le trapézoïde et le grand os. Une biopsie (B) a été faite, révélant une tumeur a cellules géantes. La patiente a bénéficié dune exérèse chirurgicale (C) de la tumeur en bloc. Les suites postopératoires étaient simples et sans complications.

**Figure 1:**
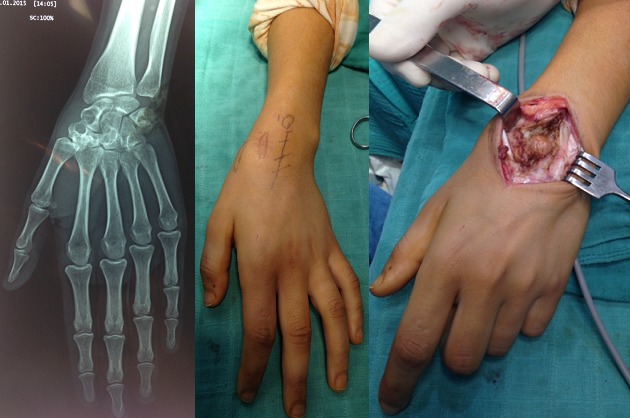
(A) radiographie du poignet de face montrant des images lacunaires au dépend du scaphoïde carpien, trapèze, trapezoide, et le grand os; (B) image clinique montrant la cicatrice de la biopsie ainsi que la voie d'abord de l'exérèse; (C) pièce opératoire

